# Factors That Influence Young Adults’ Preferences for Virtual Reality Exergames in a Weight Control Setting: Qualitative Study

**DOI:** 10.2196/58422

**Published:** 2024-12-30

**Authors:** Yanya Chen, Lina Guan, Weixin Wu, Liang Ye, Yan He, Xiaofen Zheng, Sicun Li, Bingsheng Guan, Wai-kit Ming

**Affiliations:** 1 School of Nursing Jinan University Guangzhou China; 2 Department of Infectious Diseases and Public Health Jockey Club College of Veterinary Medicine and Life Sciences City University of Hong Kong Hong Kong China (Hong Kong); 3 Department of Environmental Sciences and Engineering, Gillings School of Global Public Health University of North Carolina at Chapel Hill Chapel Hill, NC United States; 4 School of Medicine Jinan University Guangzhou China; 5 The First Affiliated Hospital of Jinan University Guangzhou Guangzhou China

**Keywords:** virtual reality, games, weight control, preferences, young adults, qualitative research

## Abstract

**Background:**

Obesity could compromise people’s health and elevate the risk of numerous severe chronic conditions and premature mortality. Young adults are at high risk of adopting unhealthy lifestyles related to overweight and obesity, as they are at a phase marked by several significant life milestones that have been linked to weight gain. They gain weight rapidly and excess adiposity mostly accrues, compared with middle-aged and older adults when weight stabilizes or even decreases. Virtual reality exergames have the potential to increase physical activity in people’s daily lives. However, the factors that influence young adults’ preference for using virtual reality exergames for weight control remain unclear.

**Objective:**

The objective of this study is to identify, characterize, and explain the factors influencing young adults' preference for weight control using virtual reality exergames.

**Methods:**

This qualitative study used semistructured interviews. In total, 4 focus group interviews were conducted with 23 young adults aged between 18 and 25 years. The qualitative data were analyzed using the Colaizzi phenomenological methodology.

**Results:**

In total, 3 major factors were found to influence young adults’ preference for virtual reality exergames in weight control settings: individual factors, social or environmental factors, and expectations of virtual reality exergames. Individual factors included experience with previous weight control methods, previous experience with virtual reality, psychological status, attitudes toward personal BMI, preference for exercise type, and acceptance of virtual reality exergames. Social or environmental factors included social definitions of beauty, weather or public health events, and knowledge of virtual reality provided. Expectations of virtual reality exergames included cost of the device, the fun of virtual reality exergames, supervision, modality of virtual reality exergames, feedback after exercise, convenience to use, and weight loss effect.

**Conclusions:**

Young adults take various factors into account when deciding whether to use virtual reality exergames for weight control. These factors can inform the development and further refinement of devices, guides, and policies related to virtual reality exergames for controlling weight.

## Introduction

Obesity could compromise people’s health and elevate the risk of numerous severe chronic conditions and premature mortality [1]. An estimated 5 million people died in 2019 from noncommunicable diseases caused by higher-than-optimal BMI, including diabetes, cancer, cardiovascular disease, digestive disorders, neurological disorders, and chronic respiratory diseases [2,3]. Besides serious threats to people’s health, it was predicted that the global costs of overweight and obesity would reach US$ 3 trillion per year by 2030 and will exceed US$ 18 trillion by 2060 if no action is taken [4]. Over the last 2 decades, obesity prevalence has doubled worldwide, with 43% (2.5 billion) of adults aged 18 years and older being overweight and 16% (890 million) being obese in 2022 [5]. Especially, young adults (aged 18-25 years) are at high risk of adopting unhealthy lifestyles leading to overweight and obesity. They are at a phase marked by several significant life milestones that have been linked to weight gain. These milestones include embarking on a college education, entering into marriage, and initiating the journey of starting a family [6]. At the young adult stage, adults gain weight rapidly and excess adiposity mostly accrues, compared with middle-aged and older adults when weight stabilizes or even decreases [7]. According to the report of the World Obesity Federation, the decrease in physical activity and a lack of exercise have contributed to a global rise in overweight or obesity [8,9]. In 2016, the prevalence of insufficient physical activity increased steadily and reached 28.5%, and over a quarter of adults (27.5%) were insufficiently physically active [10]. Global physical activity targets in 2025 (reducing insufficient physical activity by 10%) will not be achieved if current trends continue [10]. In order to increase population levels of physical activity, measures and policies must be urgently prioritized and scaled up.

The term exergames (a portmanteau of “exercise” and “games”) referred to technology-based physical activities where people could move their entire bodies for the purpose of playing [11]. As an emerging field in technology, virtual reality had attracted attention for its ability to enhance participation [12]. It allowed users to gain real-world experiences by interacting with it in a similar way to what they would experience in the real world [13]. Although the definition of exergames was different from that of virtual reality, there was an overlap between them for health [14]. That was to say, some exergames could consist of virtual reality content, while some did not [15]. These years have seen the popularity, acceptance, affordability, and accessibility of some commercial virtual reality exergames, including Nintendo Switch, Microsoft Xbox Kinect, and Wii sports [16]. Virtual reality exergames could create a presence where the users could be fully immersed in the virtual environment. Compared with traditional aerobic exercise, the adherence to playing virtual reality exergames was reported to increase by 30% in terms of attendance at physical activity, emphasizing the potential of virtual reality exergames to motivate individuals with overweight to participate in positive activities more regularly [17]. Besides promoting light- to moderate-intensity physical activity, virtual reality exergames could even be used as an effective exercise strategy for meeting physical recommendations [18]. Currently, virtual reality exergames were reported to help Parkinson’s disease patients to increase their balance skills and individuals with chronic neck pain to improve their depression and pain interference [19,20]. They have also been used for overweight and obese people, demonstrating the effect of decreasing BMI among overweight middle-aged women and increasing energy expenditure in children and adolescents with overweight or obesity [12,21]. However, to our knowledge, systematic factors that influence young adults’ preferences for virtual reality exergames in a weight-control setting remain unclear. To develop and further refine the devices, guides, and policies for weight control–related virtual reality exergames, we need to better understand the factors that influence young adults’ preferences. Hence, this study aimed to identify, characterize, and explain the factors influencing young adults' preference for virtual reality exergames in weight-control settings through a qualitative study.

## Methods

### Participants and Study Design

This qualitative study was conducted on young adults from a community in Guangzhou (Canton), making sure that their environmental factors and sociocultural backgrounds are relatively consistent. A convenience sample of young adults was used due to the small number of participants available. The inclusion criteria were shown as follows: (1) the participants were required to be within the age range of 18-25 years, (2) not having a self-reported diagnosed physical or mental disease, and (3) voluntary participation. Recruitment began with posting the advertisement on the WeChat Official Account Platform. Then, interested potential participants get in touch with us by scanning the QR code on the advertisement to add our principal investigator on WeChat. Invitation letters were sent to potential participants who provided contact details and met the inclusion criteria, explaining the purpose of the study and asking if they would like to participate. Finally, 23 young adults (ie, 12 male and 11 female) fulfilled the inclusion criteria and took part in the study. Participants received a certain amount of honoraria (50 RMB [US $6.85] per individual) after the interview.

Compared with one-on-one interviews, focus groups are premised on the assumption that group interactions facilitate the expression of interviewees' viewpoints, as participants tend to feel safer and less apprehensive when discussing challenging experiences [22]. Hence, since focus group interviews can provide a more in-depth understanding of one’s feelings or views regarding a particular topic, they were adopted to explore the factors that influenced young adults’ preferences in this study. Based on previous literature [22], the number of participants in focus group interviews was typically 5-6 participants.

### Ethical Considerations

Ethical approval was obtained from the institutional review board of City University of Hong Kong (reference number: HU-STA-00001075). Participation was strictly voluntary. Interviews were conducted only after participant consent had been obtained. The purpose of the research, the methodology, and the procedures involved were explained to the participants. Without any setbacks, participants could withdraw at any time. Additionally, we protected the participants' privacy with strict confidentiality measures. The information of participants was anonymized during transcription and only accessible to authorized members of the research team.

### Data Collection

Data were collected in September and October 2023. Individuals interested in participating in the study were required to contact the principal investigator to arrange for data collection. Qualitative data collection was conducted in a quiet meeting room at a university conveniently located near the participants' community. Participants sat comfortably in a circle so that facial expressions and body posture could be used to convey information. Each participant was informed that they could withdraw at any time. Focus group interviews were conducted using the Mandarin language in a semistructured interview format and recorded (the guiding questions are listed in Table 1). The interview comprised 2 distinct sections. During the initial section, participants explained their perceptions of previous weight control methods and virtual reality. In the subsequent section, the researchers offered a concise summary of the present situation of virtual reality exergames in the realm of weight management, ensuring that all participants attained a fundamental and comprehensive understanding. Respondents could then articulate their expectations and ideas about the application of virtual reality in weight control programs through conversations. As the data had reached saturation, we decided not to recruit other participants after interviewing 23 participants. Each interview lasted approximately 30 minutes and was video recorded and transcribed by a professional transcription service.

**Table 1:** Focus group outline.

Part	Number	Question
1	1	Please share your feelings about previous weight control methods.
1	2	Have you experienced something related to virtual reality?
2	1	If virtual reality exergames could be used to control weight, would you like to try it?
2	2	If virtual reality exergames could be used to control weight, what factors would you deem important?
2	3	Do you have any suggestions on when virtual reality exergames will be used to control weight?
2	4	Is there any additional information you wish to include in the interview content?

### Data Analysis

The Colaizzi phenomenological methodology and NVivo (version 14; Lumivero) software were adopted to analyze the qualitative data. The Colaizzi analytical approach encompassed seven distinct steps: (1) understanding the content and significance of each transcript, (2) identifying noteworthy or key from the narrative, (3) deriving or building significance from serious statements, (4) dividing explicit meanings into thematic clusters, (5) providing a detailed depiction of the phenomenon, (6) identifying the potential framework for this phenomenon, and (7) offering exhaustive descriptions to the participants. The findings of the paper were presented in accordance with the COREQ (Consolidated Criteria for Reporting Qualitative Research) to guarantee transparency and reproducibility of our methods.

## Results

### Overview

This study involved 23 participants (ie, 12 male and 11 female) aged 18-25 years (Table 2). The focus group interviews were conducted with 4 separate groups of participants. In total, three main themes emerged: (1) individual factors, (2) social or environmental factors, and (3) expectations of virtual reality exergames. These main themes were further categorized into 16 subthemes (Table 3).

**Table 2:** Characteristics of included young adults (N=23).

Characteristics		Values
Age (years), mean (SD)		21.04 (1.988)
BMI (kg/m^2^), mean (SD)		25.97 (4.244)
**Gender, n (%)**		
	Male	12 (52.2)
	Female	11 (47.8)
**Setting, n (%)**		
	Rural	12 (52.2)
	Urban	11 (47.8)

**Table 3:** Themes and subthemes.

Theme	Subtheme
Individual factors	• Experience with previous weight control methods • Previous experience with virtual reality • Psychological status • Attitudes toward personal BMI • Preference of exercise type • Acceptance of virtual reality exergames
Social or environmental factors	• Social definition of beauty • Weather or public health event • Knowledge of virtual reality provided
Expectations of virtual reality exergames	• Cost of the device • Fun of virtual reality exergames • Supervision • Modality of virtual reality exergames • Feedback after exercise • Convenience to use • Weight loss effect

### Individual Factors

#### Experience With Previous Weight Control Methods

Previous weight control methods included diet, exercise, physical therapy, and traditional Chinese medicine. Some young adults were confused as they could not find a suitable weight loss method. Several participants had difficulty keeping the weight off. Exercise as a method of weight control was tedious, and it was difficult to participate in others' “sports teams suddenly” sometimes.

I feel like every sport has a threshold. Picking up a new sport, such as badminton and basketball... can lead to difficulty integrating myself into other experienced players.

Dancing to web-based videos can be dull, but to control my weight, I would endure the boredom. If possible, I would choose more interesting ways of exercising.

#### Previous Experience With Virtual Reality

Approximately 50% of interviewees had experienced virtual reality before, and most participants preferred to use virtual reality exergames to control weight since they regarded it as interesting, immersive, and engaging. After experiencing it, a minority of participants considered virtual reality exergames uncomfortable and dizzy, so they lost interest.

I experienced virtual reality in science museums, viewing snowy mountain scenery from a high altitude with virtual reality glasses. I was engrossed in the novelty. Perhaps it would be more appealing to control weight via VR than outdoor sports.

Now it's (virtual reality) quite realistic. I played a similar roller coaster simulation game before. It simulates the perspective of you sitting on a roller coaster. Although you don't feel the actual weightlessness, when it rushes down, it still feels quite realistic…very willing to try (virtual reality exergames)

#### Psychological Status

Some young adults were perceived as introverted and unwilling to go out. Therefore, they were partial to exercise at home, adopting virtual reality exergames. Some young adults with large BMI, low self-esteem, and sensitivity favored controlling weight at home through virtual reality exergames, avoiding encountering acquaintances in exercise procedures.

Some people like me simultaneously possess a social butterfly identity online and a socially awkward persona in real life…we feel comfortable approaching strangers in video games and becoming acquainted virtually. However, if we encounter someone on the nearby treadmill in a gym, it is impossible for us to walk up and start a conversation. I prefer online workout buddies as long as our relationship serves to motivate exercising.

I'm an ‘i-person’ (introverted person). I just don't like going out. Virtual reality exergames feel more inclusive for people like us.

#### Attitudes Toward Personal BMI

Some young adults believed their weight was acceptable or did not care about others’ comments about their figures. Accordingly, there was no intense aspiration to use virtual reality exergames to control weight.

I have a ‘let it be’ mentality…Regardless of my size, this is my body… I do not have a strong motivation for weight intervention except it affects my health…whether through virtual reality exergames or outdoor activities.

I guess others might have negative opinions about me, but I don't care. If there are virtual reality exergames, I'll use them; if not, it's no big deal.

#### Preference of Exercise Type

Currently, virtual reality exergames offer a wide range of sports options, encompassing football, badminton, fencing, and dancing. However, the type of physical activity could influence young adults' usage and adherence to virtual reality exergames for weight management. For instance, female-identified persons preferred dance-based games within the virtual reality exercise realm while disinterested in excessively aggressive game genres. On the other hand, male-identified persons leaned toward ball games or fencing as their preferred forms of virtual reality exergames. Nevertheless, both genders expressed reluctance toward using virtual reality glasses for exercising through virtual reality exergames.

The duration of applying virtual reality exergames can last for one month or year… depends on if we are entertained by the type of game and exercise.

#### Acceptance of Virtual Reality Exergames

More than half of the young adults were willing to adopt virtual reality exergames for weight control, although a few participants did not agree that it had good results due to poor virtual reality experience and other reasons. Participants with a positive attitude believed that virtual reality exergames could motivate people to exercise more consistently by offering a variety of exercise options and fun experiences. They also found that these games were challenging enough to leave them feeling fatigued after working out. This supportive attitude would provide a basis for young adults to use virtual reality exergames for weight control.

In fact, the greatest challenge about weight control is persistence. As long as the game is well designed, engaging and immersive, surely it will be a boost for those people having trouble sticking to their workouts, so why not opt for such a form of exercise?

### Social or Environmental Factors

#### Social Definition of Beauty

Almost all interviewees agreed that society defined beauty as being slender. This popular aesthetic trend became a driving force for some people to exercise, making them willing to try a new exercising way that was easier to stick to and had fun.

Clothes sizes are also designed smaller and smaller these days, guiding people to standardize beauty with slenderness. In order to look more attractive (slim), I have to exercise, and the virtual reality exergames can be an option for me.

Usually, because we study in Guangdong (Canton), there are many local students here, and indeed, the girls from Guangdong are generally petite and very slim. Standing next to them, I can't help but feel a bit envious... I still want to try various weight control methods as long as they are effective.

#### Weather or Public Health Event

Many interviewees said that when they encountered weather conditions such as rain, cold winters, and public health events such as the COVID-19 pandemic, which prevented people from going out to exercise, they would choose the virtual reality exergames to exercise at home.

During COVID-19, I was required to quarantine at a hotel for 14 days and at home for another 7 days, and because of the lack of exercise and the greasy meals, I gained weight very quickly, but I was not allowed to get out of the house, and at that time I was thinking how nice it would be to have a set of consoles such as the Nintendo, that would be good to both kill time and control my weight.

#### Knowledge of Virtual Reality Provided

About half of the interviewees were unclear about the definition of virtual reality, not to mention how exercise was done through virtual reality. In total, 4 interviewees said that they usually did not have access to knowledge related to virtual reality and did not understand the development of virtual reality, so they hesitated to use the virtual reality exergames for weight control.

I don’t know much about new technology like virtual reality, ...... also not interested in virtual reality exergames.

For new technologies like this (virtual reality), I feel that there hasn't been a widespread way to access them in the past. Currently, most of the promotion focuses on exercise, and short videos make it quite impactful. So, it seems that diet and exercise are still the main methods.

### Expectations of Virtual Reality Exergames

#### Cost of the Device

For young adults, their income was not high; most of them were students and still received living expenses from their parents. Some interviewees knew that some interesting virtual games could be used for exercise and weight control. However, they could not afford expensive virtual gaming equipment. Therefore, they said that they would only favor exercising in this way if the equipment cost could be a bit more affordable.

As a junior in college, I get all my living expenses from my parents. Therefore, I don't want to buy expensive gaming equipment, even though it might help me lose weight. I can just run a few laps on the track field by myself.... I would only choose to use it if (the gaming device) was cheaper.

#### Fun of Virtual Reality Exergames

The fun of virtual reality exergames was one of the important factors influencing young adults’ choice of weight control measures. Most participants highlighted that the gratifying and engaging nature of these games would play a role in maintaining their engagement. However, the fun aspect tends to diminish if players are restricted to a single game type for too long, particularly when it ceases to offer new challenges.

In team sports such as volleyball and bowling... a lack of strong interaction or a failure to meet expectations could lead to infrequent participation.

#### Supervision

For the majority of interviewees, supervision provided by virtual reality exergames was essential in managing weight. They were more likely to frequently use these games under supervision, but in its absence, their commitment tends to wane.

Given specific daily check-in tasks, I'm likely to adhere to them; however, in the absence of such directives, I tend not to engage in virtual reality exercises at home.

If there's no check-in or supervision, you just won't use it.

#### Modality of Virtual Reality Exergames

Most interviewees revealed dislike for exercising alone, emphasizing their commitment to exercise increases with the presence of family or friends. This sentiment held true for managing weight through virtual reality exergames, where they valued the companionship of family and friends.

Nintendo offers games like badminton, which initially seemed uninteresting when I played alone…… But during the Chinese New Year festivities, engaging in competitive play with friends transformed the experience, leading me to maintain regular exercise throughout the winter break surprisingly.

#### Feedback After Exercise

Many participants hoped that the virtual reality exergames system could provide some feedback, encompassing win-loss records, heart rate, blood pressure, etc. Being aware of the positive impact of their exercise or favorable game results, a portion of these individuals might experience a sense of success.

Receiving feedback is undeniably better than not getting any... Witnessing my heart rate rise in the workout summary assures me that I'm actually exercising.

This (virtual reality exergames) needs to provide feedback after exercise for me to use it. Otherwise, without any kind of reference point, I wouldn't know how much or how well I'm exercising.

#### Convenience to Use

In total, 3 participants mentioned that the user-friendliness and portability of the gaming system influenced their inclination to use virtual reality exergames.

I lean towards more compact models, which allow me to easily take the equipment along even when I'm traveling for a few days.

I might start by trying it out... Also, the convenience of the equipment is important; otherwise, it would be difficult for me to stick with using it(virtual reality exergames) to control my weight.

#### Weight Loss Effect

In total, 7 interviewees noted that success in weight loss impacts their choice to use virtual reality exergames for weight management. This is especially significant for those with a higher BMI, for whom the effectiveness in controlling weight is a major factor.

If weight loss is ineffective, the VR game is mere entertainment, lacking its promised weight reduction functionality. My initial purpose for buying the game was to sustain a regular exercise routine. It feels like a waste of money if it fails to give significant results.

## Discussion

### Principal Findings

This qualitative study identified, characterized, and explained the factors influencing young adults' preference for weight control using virtual reality exergames. Through interviewing 23 young adults, it was concluded that the respondents were mainly affected by individual factors, social or environmental factors, and expectations of virtual reality exercise games.

The effect of weight loss was one of the important factors affecting whether or not young adults choose virtual reality exergames for weight control. As Comeras-Chueca et al [23] reported, different types of virtual reality exergames produced different weight loss effects and were no worse than traditional exercise. Graves et al [24] examined the energy expended while playing 3 distinct Wii Sports games and demonstrated that boxing resulted in the highest expenditure of energy (4.05 kcal/min), surpassed by tennis (3.05 kcal/min) and bowling (2.75 kcal/min). Similar results also revealed that the energy expended during Dance Dance Revolution and Wii Sports boxing was comparable to that of walking at a moderate intensity (5.7 km/h) [25]. Furthermore, virtual reality exergames could be an effective way for obesity management among overweight middle-aged women, as the results of the research showed that the mean BMI significantly decreased from 26.04 to 24.65 kg/m^2^ after 8 weeks of the virtual reality exergames program [12]. Meanwhile, these virtual reality exergames aim to enhance the fun and enjoyment of physical activities by providing a virtual environment that is tailored to the preferences of young adults, especially for those with obesity and low motor skills [23]. These make those young adults feel more agile, more skilled, and more powerful, so that these young adults will have more desire and more interest in practicing other sports [23]. Therefore, the virtual reality exergames caught the attention of young adults who are reluctant to engage in traditional physical activities. That is to say, virtual reality exergames change the attitude of young adults from rejection to habit, helping them get a more active lifestyle at the same time.

The results of this study showed that most young adults preferred to use virtual reality exergames in the company of friends or family. Young adults consumed more energy when playing virtual reality exergames with a peer [26]. Second, with the engagement of friends and family members, young adults' adherence to exercise would increase. In the virtual world, the companionship of friends or family could boost individuals' perceived competency, satisfaction, and self-belief in executing group tasks [27]. The social interaction might provide feedback and recognition, resulting in a sense of satisfaction when their accomplishments are noted and appreciated by others [28]. When playing virtual reality exergames socially with peers, it was encouraged to engage in more intense and sustained physical activity throughout each gaming session, particularly in the posterior regions [29]. It was speculated that team cohesion enhanced adherence to an exercise regimen when individuals engage in physical activities as part of a group [30]. Third, social support and self-esteem increased when people worked out in teams, which served as motivation and enabled sustained physical activities [31].

The cost of the device influenced young adults’ preference for weight control using virtual reality exergames. Virtual reality exergames did not reach all overweight and obese adolescents due to their high cost; the software and hardware were expensive [32]. For example, the cost of an average head-mounted virtual reality was still relatively high (average CAD $ 585 [US$ 460]) [33], which limited the use of virtual reality exergames for weight loss in a subset of the population. Especially 18- to 25-year-old young adults who are studying or just entering work do not have too much income. However, with the growing interest in virtual reality exergames, young adults spend more and more on them. Statistics reveal that in 2017, US consumers spent a total of 29.1 billion dollars on video games, marking an increase of 10 billion dollars compared to the previous 5-year period [34]. In addition, with technology development, virtual reality exergames development costs may be getting lower and lower. The cost will not be the main factor that influences young adults’ preferences in the future.

This study had one strength of being a detailed qualitative analysis with a limited number of participants, enabling a deeper understanding of their perceptions. Through the in-depth understanding of young adults’ perspective, the results can benefit the development and further refinement of devices, guides, and policies related to virtual reality exergames for controlling weight. An additional strength of this study was using focus groups as a methodology. The direct interaction between the participants in one group enabled participants to ask the key questions and explore a variety of opinions. This study also had some limitations. First, this study may lack representativeness of the general population due to the non-random selection of participants and potential failure to capture the population's diverse characteristics. Future research should address this limitation by using larger sample sizes and mixed methods, such as surveys and focus groups. Another limitation of this study was that it was conducted in a single community and might not translate to other areas. This should be overcome by seeking variation among participants and paying attention to more general factors that can be applied to other areas in future studies.

### Conclusions

The results indicated that young adults consider complex factors when considering whether to use virtual reality exergames for weight control. These included individual factors such as the experience of previous weight control methods, previous experience with virtual reality, psychological status, etc, social or environmental factors such as the social definition of beauty, weather or public health events, etc, and expectations of virtual reality exergames. The results of this study can inform the development and further refinement of devices, guides, and policies related to virtual reality exergames for controlling weight.

## Abbreviations

COREQ: Consolidated Criteria for Reporting Qualitative Research

## Data Availability

The datasets generated during and/or analyzed during this study are available from the corresponding author on reasonable request.
